# A Comparison of Simulated and Field-Derived Leaf Area Index (LAI) and Canopy Height Values from Four Forest Complexes in the Southeastern USA

**DOI:** 10.3390/f9010026

**Published:** 2018

**Authors:** John S. Iiames, Ellen Cooter, Donna Schwede, Jimmy Williams

**Affiliations:** 1 U.S. Environmental Protection Agency, National Exposure Research Laboratory, Exposure Methods and Measurements Division, 109 T.W. Alexander Drive, Research Triangle Park, NC 27711, USA; 2 U.S. Environmental Protection Agency, National Exposure Research Laboratory, Atmospheric Modeling and Analysis Division, 109 T.W. Alexander Drive, Research Triangle Park, NC 27711, USA; 3 Texas A & M University, Agri-Life Research, Temple, TX 76502, USA

**Keywords:** LAI, leaf area index, EPIC, simulation, satellite, MODIS, biomass, evaluation, southern U.S. forests

## Abstract

Vegetative leaf area is a critical input to models that simulate human and ecosystem exposure to atmospheric pollutants. Leaf area index (LAI) can be measured in the field or numerically simulated, but all contain some inherent uncertainty that is passed to the exposure assessments that use them. LAI estimates for minimally managed or natural forest stands can be particularly difficult to develop as a result of interspecies competition, age and spatial distribution. Satellite-based LAI estimates hold promise for retrospective analyses, but we must continue to rely on numerical models for alternative management analysis. Our objective for this study is to calculate and validate LAI estimates generated from the USDA Environmental Policy Impact Climate (EPIC) model (a widely used, field-scale, biogeochemical model) on four forest complexes spanning three physiographic provinces in Virginia and North Carolina. Measurements of forest composition (species and number), LAI, tree diameter, basal area, and canopy height were recorded at each site during the 2002 field season. Calibrated EPIC results show stand-level temporally resolved LAI estimates with *R*^2^ values ranging from 0.69 to 0.96, and stand maximum height estimates within 20% of observation. This relatively high level of performance is attributable to EPIC’s approach to the characterization of forest stand biogeochemical budgets, stand history, interspecies competition and species-specific response to local weather conditions. We close by illustrating the extension of this site-level approach to scales that could support regional air quality model simulations.

## 1. Introduction

The status and dynamics of vegetation leaf area, often reported in terms of leaf area index (LAI), can be a critical determinant of regional air and water quality [1]. LAI is commonly used as a surrogate of photosynthetically active area when photosynthesis is the principle process controlling chemical exchange between the atmosphere and underlying land surfaces. Leaf area influences the sequestration of carbon from carbon emissions [2], the removal of pollutant species through deposition [3], the biogenic emission of volatile organic compounds (BVOC) that contribute to tropospheric ozone formation [4], and the emission of greenhouse gases [5]. Temporally resolved leaf area and canopy heights for natural forest stands are critical inputs for process-based meteorological models. Leaf area estimates contribute to the calculation of surface evapotranspiration and albedo, and canopy height largely determines surface roughness, which contributes to mechanical mixing of the atmosphere [6]. Calculation of gaseous air pollutant deposition velocity (V_d_) frequently requires values for leaf area and surface roughness [7–9]. The Clean Air Status and Trends Network (CASTNET), for instance, measures atmospheric concentrations and then estimates water vapor, ozone (O_3_), sulfur dioxide (SO_2_), and nitric acid (HNO_3_) fluxes using the Multilayer Model (MLM) [8,10,11]. The MLM inputs a generalized annual LAI time-series developed from measured values of maximum LAI for each plant species and typical phenology. Estimates of deposition velocity calculated by MLM were seen to be highly sensitive to LAI time-series parameters [12] with differences in V_d_ of about 25% for sulfur dioxide and nitric acid and greater than 60% for ozone. On a regional scale, the United States Environmental Protection Agency’s (US EPA) Community Multiscale Air Quality Model (CMAQ) [8] relies on output from the Weather Research Model (WRF) [13] and the Pleim-Xiu (PX) land surface scheme [14]. PX LAI estimates are based on deep soil temperature, an LAI response function based on soil temperature and a specified minimum and maximum LAI for each land use classification which results in known biases [15].

LAI inputs into air quality applications that rely on model estimates of chemical flux include periodic in situ point sampled and Light Detection and Ranging (LIDAR) LAI measurements, static look-up values, and satellite-derived LAI values. A number of studies have reported that simulating both accurate leaf- and canopy-scale fluxes is not possible when leaf-scale fluxes are scaled using an inaccurate LAI [16–19]. While more temporally and spatially detailed LAI inputs are likely to improve model estimates of meteorological conditions, biogenic emissions, and O_3_ precursor estimates, interspecies competition, age and spatial distribution make temporally resolved LAI estimates for minimally managed or natural forest stands particularly difficult to develop. Satellite-based LAI estimates hold promise for retrospective analyses, e.g., [20,21], but we are still learning how best to make use of these data, and they will never be available for future meteorological alternative management applications. Therefore, we must continue to supplement temporally resolved, remotely sensed vegetative leaf area estimates with numerical model estimates.

The objective of this study is to evaluate the capacity of a biogeochemical agroecosystems model to generate more biologically (process) representative, accurate and temporally resolved forest stand-level LAI estimates than existing numerical methods. We calibrate the USDA Environmental Policy Integrate Climate (EPIC) model from LAI estimates evaluated at four mixed forest stands in the southeastern U.S. We then demonstrate the practical areal application of this approach to represent mixed-age, mixed forest stands that could support nitrogen flux estimates for air quality assessment and supplement remotely sensed observations.

## 2. Materials and Methods

### 2.1. Site Descriptions

**Figure 1:**
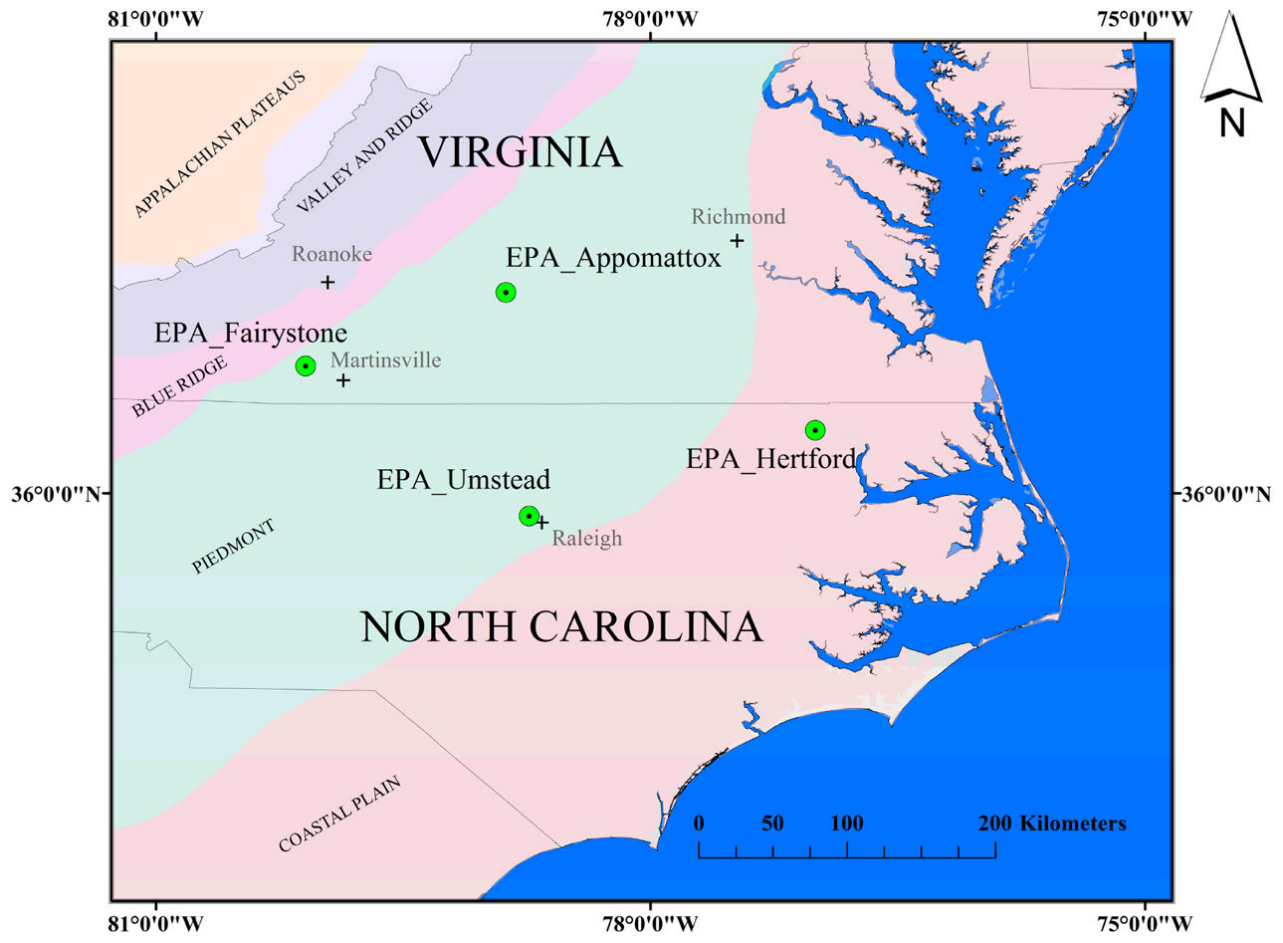
Four LAI field site locations in Virginia and North Carolina, USA.

The near-lab study site for the US EPA Office of Research and Development is the Albemarle-Pamlico Basin (APB), located in central-to-northern North Carolina and southern Virginia. The APB has a drainage area of 738,735 km^2^ and includes three physiographic provinces: mountain, piedmont and coastal plain, ranging in elevation from 1280 m to sea level. The APB sub-basins include the Albemarle-Chowan, Roanoke, Pamlico, and Neuse River basins; all draining into the second largest estuarine system within the continental United States. Four 1 km^2^ LAI calibration/validation sites were chosen within the APB representing all three physiographic provinces: 1. Appomattox (Upper Piedmont), 2. Hertford (Coastal Plain), 3. Fairystone (Upper Piedmont-Mountain), 4. Umstead (Lower Piedmont) (Figure 1). General descriptions of these sites are provided below.

#### 2.1.1. Appomattox

The Appomattox field site is located in Campbell County, Virginia (37.219°N, −78.879°W) approximately 15.5 km south-southwest of Appomattox, Virginia (Figure 1 and Table 1). This Upper Piedmont region has a range in elevation from 165 m to 215 m above mean sea level. The area is a mixture of rural agricultural fields and managed (Loblolly pine—*Pinus taeda*) and unmanaged (oak-hickory) forest stands. The MeadWestvaco Corporation, a supporter of the Sustainable Forestry Initiative, permitted sampling access to the US EPA for LAI research on 505 hectares in 2002. Land cover (LC) percentages within the 1 km^2^ area include: (1) coniferous (Total—67.6% (unthinned—30.6%, thinned —37.0%)); (2) deciduous (21.7%); and (3) other vegetation (harvested—10.6%). The modeled and in situ LAI comparison uses the 30.6 hectare unthinned pine stand; a 100% planted loblolly pine dominant-codominant crown class (15.9 m canopy height) underlain with a variety of understory deciduous species (Supplementary Materials Figures S1 and S2 and Table S1). The dominant soil types consist of very deep, well-drained, moderately permeable soils (Georgville and Tatum loams) on 2% to 15% slopes.

#### 2.1.2. Hertford

The Hertford site is located in Hertford County, North Carolina (36.383° N, −77.001° W), approximately 5.8 km west-southwest of Winton, North Carolina (Figure 1). This coastal plain site is 8–10 m above mean sea level (0% slope) with a moderately well drained thermic Aquic Hapludult soil type (Craven fine sandy loam). This site is dominated by 19 year-old planted loblolly pine, and was managed by International Paper, Inc. LC percentages within the 1 km^2^ area include: (1) coniferous (72.3%); (2) deciduous (17.8%); and (3) other vegetation (agriculture—9.9%). The modeled and in situ LAI comparison was done only within the 19 year-old managed pine stand (Supplementary Materials Figures S3 and S4 and Table S1).

#### 2.1.3. Fairystone

The Fairystone site is located in both Henry and Patrick Counties in Virginia (36.771° N, −80.092° W), approximately 20.7 km west-northwest of Martinsville, Virginia (Figure 1). This Blue Ridge eastern foothills site is 409 m above mean sea level and is defined by well drained mesic Typic Hapludult soil types (Fairystone and Littlejoe Series). The Virginia Department of Inland Game and Fisheries manage this upland hardwood site (100.0% deciduous) maintaining habitat suitable for turkeys, deer and a variety of small game and nongame wildlife (Supplementary Materials Figures S5 and S6 and Table S1).

#### 2.1.4. Umstead

The North Carolina Parks and Recreation Department manages the Umstead site, which is located 12.5 km northwest of Raleigh, NC (William B. Umstead State Park) (Figure 1). This 2258-hectare park is a mixture of mature deciduous and coniferous tree species, with an average age approximately 75–85 years old (Supplementary Materials Figures S7 and S8 and Table S1). This Piedmont site ranges in elevation between 75.3 m and 120.5 m above mean sea level (*x* = 94.0 m), with slopes ranging between 0.1% and 25.0% (*x* = 6.2%) and is defined by 13 variant sandy loam soil types. Land-cover percentages within the 1 km^2^ area include: (1) deciduous (77.8%) and (2) coniferous (22.2%) forest types.

### 2.2. In Situ Measurements

**Figure 2:**
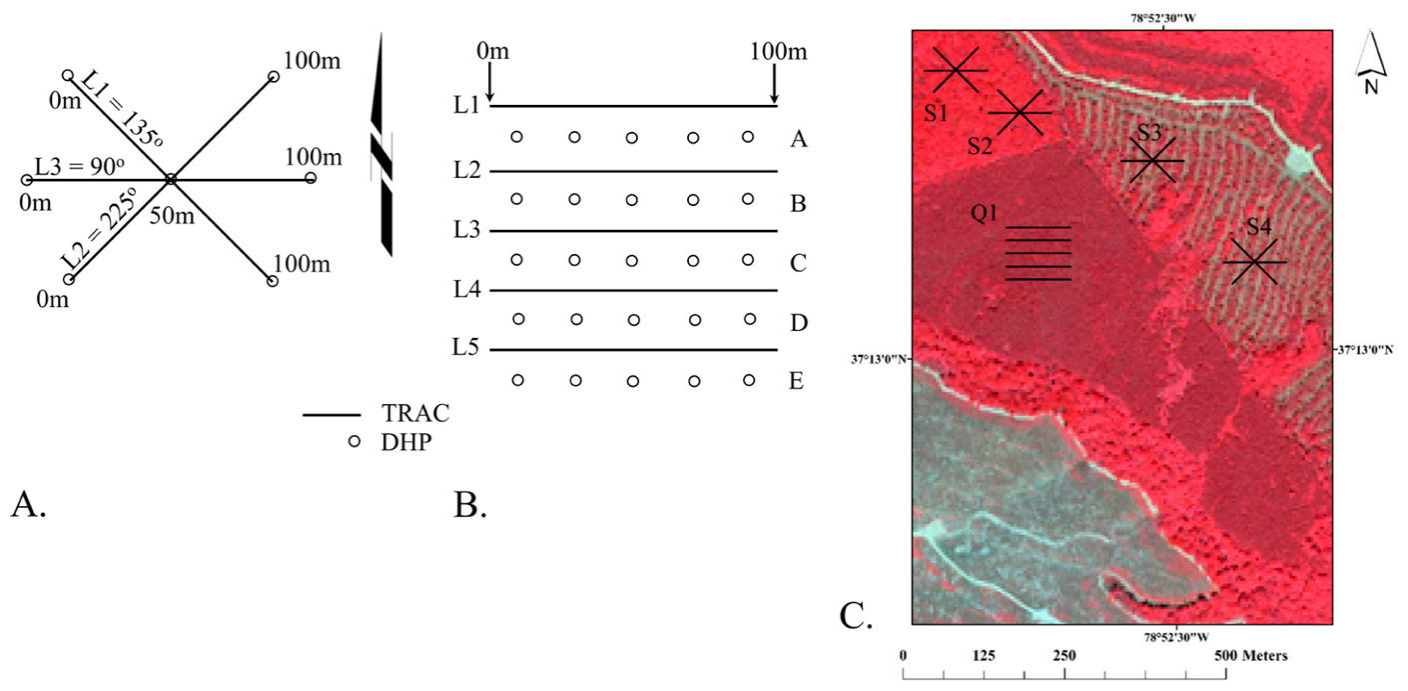
Plot design for (**A**) 50-m or 100-m sub-plot and (**B**) 100 m × 100 m quadrat. Distribution of quadrat and sub-plots on Appomattox research site (**C**) is illustrated by Q1 and S1–4. Note: DHP—Digital Hemispherical Photography; TRAC—Tracing Radiation and Architecture of Canopies analyzer.

Forest stand characteristics were collected for all four validation sites over the 2002 growing season including (1) species type, and (2) structural (crown closure, height, diameter, LAI) and (3) stocking attributes (basal area and trees per hectare). On each site, the sampling design includes the establishment of the primary sampling unit, the quadrat (Figure 2B). Each quadrat is a 100 m × 100 m grid delineated by five parallel 100 m east-to-west (E–W) oriented transects labeled L1–L5—each transect line is separated 20 m apart in a north south (N–S) direction, with L1 representing the northern-most transect. Interspersed between these transects are 25 ‘point sampling’ locations in a N–S and E–W grid network, separated 20 m apart, with the northwestern most point established between L1 and L2 transects at a 10-m inset from the origin of L1 and L2. The secondary sampling unit was three intersecting 50 m or 100 m transects at 90°, 135°, and 225° (Figure 2B). These sub-plots were located to augment data collected on the primary sampling unit(s), the quadrat (Figure 2C). Only one quadrat (Q1) was laid-out on three of the four research sites (Appomattox, Hertford, and Umstead), with four quadrats (Q1–Q4) established on the Fairystone site. Quadrat replicates were applied only at Fairystone to account for terrain and aspect differences prevalent in this upper piedmont region. Sub-plots were applied to account for multiple forest types within 1-km area (i.e., Appomattox).

Measurements of the forest structural attributes height (m) and diameter (cm) were made at all sites within the 100 m × 100 m quadrat areas using a point sampling method using a basal area 10-factor prism for trees larger than 5 cm in diameter at breast height (DBH), with breast height 1.37 m above the base of the tree. Stocking values, expressed as trees per hectare (TPH) and basal area per hectare (BA/H) were calculated from the point sampling data. BA is the cross-sectional area of a tree at 1.37 m above the tree base per unit area [22]. Three plots within each quadrat were sampled for understory components (stems less than 5 cm DBH) using a 4.57 m radius fixed area sampling method. Canopy closure, defined as the percent obstruction of the sky by canopy elements, was estimated using a Geographic Resources Solutions (GRS) Densitometer (http://www.BenMeadows.com/) along transects 1, 3, and 5 in each quadrat, taking obstruction/no obstruction readings by canopy height class (i.e., understory, intermediate, dominate) every two meters.

We define LAI here as one-half the total green leaf area per unit ground surface [23]. LAI estimates are based on the indirect in situ optical LAI estimation technique combining measurements from the Tracing Radiation and Architecture of Canopies analyzer (TRAC) optical sensor and digital hemispherical photography (DHP) in deciduous and coniferous forest stands. Parameters assessed within the TRAC-DHP method included the element clumping index (Ω_E_) derived from the TRAC instrument, the effective leaf area index (L_e_) measured with DHP, and the needle-to-shoot area index (λ_E_) and the woody-to-total index (α) measured in the field and in the laboratory. LAI calculations use these characteristics as inputs into the modified Beer-Lambert light extinction function [24]:

(1) $$\text{LAI}=(1-\alpha )\times [L_{e}(\lambda_{E}/\Omega_{E})]$$

The TRAC instrument records downwelling solar photosynthetic photon flux density (PPFD) through the canopy during direct light conditions in order to measure foliage clumping at scales larger than the shoot. TRAC is sampled along all five line transects within each quadrat and along one of three sub-plot line transects, dependent on the line transect closest to solar perpendicularity —measurements are made at a solar angle between 30° and 60° [24]. DHP measurements were derived from a Nikon CoolPix 995 digital camera with a Nikon FC-E8 fish-eye converter in diffuse light conditions in order to extract the effective LAI or also known as plant area index (PAI). DHP measurements were made at the 10-, 50-, and 90-m marks along lines A, C, and E within each quadrat and were made during diffuse light conditions (at dawn or dusk). TRAC and DHP measurements were recorded across all four sites for the following dates: Fairystone (1 May 2002, 25 June 2002, 8 July 2002, 4 September 2002), Appomattox (6 March 2002, 23 May 2002, 30 July 2002), Umstead (13 April 2002, 23 April 2002, 21 October 2002), and Hertford (5 March 2002, 9 April 2002, 18 June 2002). Processing of TRAC data using TRACWin 3.7.3 software [24,25] and DHP measurements make use of Gap Light Analyzer (GLA) software [26].

### 2.3. Soil and Meteorology

**Figure 3:**
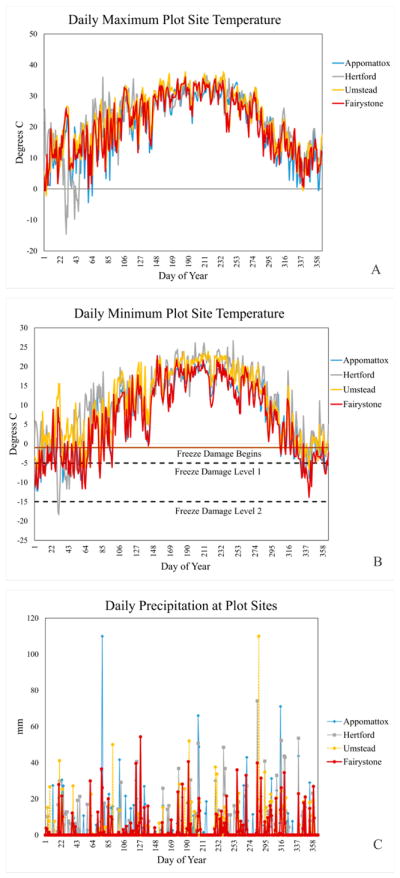
Daily weather at the four NCEI Coop locations representing the year 2002 forest plot sites (**A**) maximum temperature (°C), (**B**) minimum temperature (°C), and (**C**) precipitation (mm). The dashed lines in Figure 3B define two points on the frost damage function curve (see discussion in Section 2.4 and Supplementary Materials Data Part 3).

Biogeochemical models usually require detailed multilayer soil profiles as simulation inputs. This information was not available for the field study sites, and so we used a representative soil profile at all locations (Supplementary Materials Table S2). Historical weather data from the nearest National Oceanographic Atmospheric Administration’s (NOAA) National Centers for Environmental Information (NCEI) Cooperative Observing Station (Coop) to the forest observation site (Stand Site) were selected (Table 1). Figure 3 shows daily time series of maximum temperature, minimum temperature and precipitation at these locations during 2002.

### 2.4. The EPIC Model

The simulation of forests in EPIC is adapted from methods pioneered in the Agricultural Land Management Alternatives with the Numerical Assessment Criteria (ALMANAC) model [27]. Changes within this general plant model were developed to better address forest species [28]. Forest regrowth in Canadian Boreal forest ecosystems following disturbances such as forest fires, clear cuts and insect infestations is detailed in a boreal forest version of ALMANAC (ALMANACBF) [29]. Much of this research was performed to improve agro-forest representation in the Soil Water Assessment Tool (SWAT) [30] that characterizes the forest stand as a distinct land unit of a single or consistent combination of tree species of similar age and productivity [31,32]. The EPIC implementation of this research lacks some, and simplifies other ALMANAC processes so that EPIC and ALMANAC simulation results for the same forest ecosystem may be similar, but they will not necessarily be identical.

EPIC is a hybrid, process-based, semi-empirical biogeochemical model characterizing field-sized areas up to about 100 ha [33]. Previous EPIC applications deal primarily with managed agricultural crops, but a few studies consider natural and managed shrubland and forests. EPIC was used to evaluate light interception as a predictor of water use in hedge intercrop, monocrop, and hedge monoculture (*Senna spectabilis cv. Embu*) systems in a semi-arid environment in Kenya [34]. A spatially extended version of EPIC, The Agricultural Policy/Environmental Extender (APEX) was examined to see if it could replicate the effects of silvicultural practices on streamflow and loading of sediments and nutrients in nine small Texas watersheds [35] (http://blackland.tamu.edu/models/apex). In another EPIC application, researchers returned to the same East Texas watersheds to explore N, P and herbicide losses across three undisturbed control watersheds, three conventional clearcut watersheds and three intensive clearcut watersheds [36]. None of these studies fully explores EPIC model performance for forest stand LAI and canopy height. We hypothesize here that the EPIC implementation may be sufficient for the regional-scale air quality applications suggested in the introduction.

The most critical aspects of EPIC for the present study include the calculation of LAI, the characterization of multiple, competing species, and canopy height. For a particular species, potential values of leaf expansion, final LAI, and leaf duration, determined by plant species parameters and temperature, are reduced by unfavorable air temperatures, radiation, soil water, nutrient, aluminum and aeration conditions [33]. A series of s-curve functions provide daily values of (1) maximum potential LAI as a function of plant population, (2) leaf area expansion up to the maximum value for the growing season, and (3) loss of leaf area late in the season (Supplementary Materials Tables S3–S5). While perennials mature within a single growing season, multiple growing seasons are required for trees to reach maturity. In these cases, maximum daily potential LAI adjusts to include plant response to stress, and is scaled on an annual basis to reflect the time to tree species maturity (years). Other EPIC LAI adjustments reflect growth initiation in the spring and leaf freeze damage. Day length triggers leaf initialization. Once triggered, leaf expansion is driven by heat units, calculated using a species-specific base temperature. LAI reductions (i.e., leaf damage) occurs when daily minimum temperatures drop below −1 ° C. A simple exponential function of species age and height at maturity provides annual tree height.

The plant competition component of EPIC adapts expressions developed for the ALMANAC model. Up to 10 plant species can compete for light, water and nutrients. EPIC simulates light interception by leaf canopies using Beer’s law [37] and the leaf area index (LAI) of the total canopy. No regular row spacing is assumed in a natural forest stand, and so EPIC assigns the light extinction coefficient (k) a value of 0.685 for all forest stand species. Light competition is then simulated as a function of both LAI and canopy height. This is in contrast to the ALMANAC approach that uses different values of k for different species and for different row spacings [28].

EPIC multi-species simulation of tree growth and decline also responds to water and nutrient stress. The water balance consists of separate transpiration calculations for each species, with each using the water it needs if sufficient water is present in the common/shared rooting zone. The nutrient balance (N and P) allows each species to acquire sufficient nutrients to meet its demands if adequate quantities are available in the common/shared rooting zone. If nutrients or water is limited, the provisioning order rotates each day so that no single species receives a competitive advantage. Table 2 provides an example of water, nitrogen and temperature stress calculations for tree species simulated by EPIC at the Appomattox field site during 2002. In contrast, ALMANAC simulates species-specific competitive advantage for water and nutrients based on physical characteristics such as age, rooting depth and extent.

## 3. Results

### 3.1. Model Calibration

EPIC LAI calibration involves modification of distributed plant parameter values. Some of these parameter values reflect process-level understanding, field data and published literature, while others reflect species similarity. Plant parameter values for tree species added to or modified from the distributed EPIC for this study derive from information contained in the USDA Silvics manual [38] which describes each species geographic distribution, shade tolerance, maximum canopy height and seasonal growth pattern and temperature sensitivity. These initial values were then refined (calibrated) using field observations of stand-level LAI and maximum canopy height while maintaining plausible physical and process driven relationships. Table 3 provides the observation set used to calibrate the model. Section 2.3 describes soil and observed daily temperature and precipitation inputs to EPIC at the four forest sampling locations. We then let EPIC simulate values of daily average wind speed, relative humidity and radiation using statistical moments of climatological data, e.g., a statistical weather generator [33]. Long-term average nitrogen concentrations in precipitation are estimated using NADP data (http://nadp.sws.uiuc.edu). Calibration is complete when there is no further improvement in stand-level LAI correlation with observed LAI, LAI bias and canopy height with further parameter modification.

### 3.2. Calibrated Model Results

**Figure 4:**
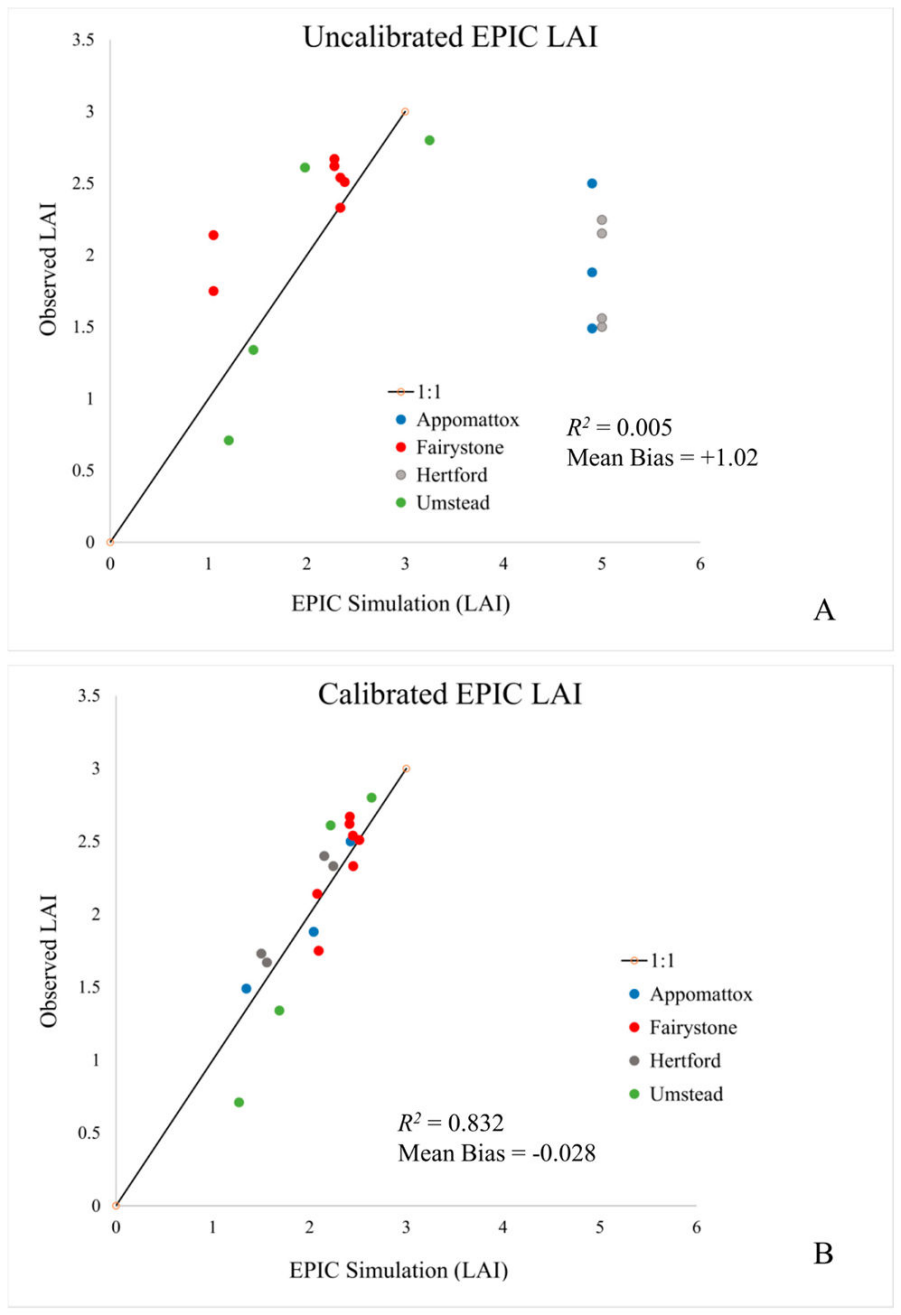
Comparison of (**A**) uncalibrated and (**B**) calibrated EPIC simulated LAI and observation.

Table 4 summarizes site species characteristics for the simulation. Species age at biological maturity is a calibrated result, but is constrained to reflect ranges reported in USDA (1990). The letter “C” indicates a species calibration site. The letter “V” indicates a species verification site. Note that data were insufficient to verify the calibration of some species. Original (uncalibrated) and final (calibrated) EPIC tree species parameter values are provided in Supplementary Materials Tables S3–S5. Figure 4 compares calibrated and uncalibrated EPIC simulation results to observed LAI values across all four sites. Calibration improves overall simulated *R*^2^ from 0.005 to 0.83 and reduces mean bias from −0.19 to 0.008. Uncalibrated model behavior at hardwood (Umstead and Fairystone) and Pine dominated (Appomattox and Hertford) sites are distinctly different. This difference relates to an unrealistically large uncalibrated value of maximum LAI, 5.0 for Loblolly Pine. When these metrics are re-calculated just for the hardwood stands, the uncalibrated correlation increases to 0.74 but the uncalibrated mean bias increases to −0.218, indicating the need for additional calibration even for hardwood-dominated stands.

Overall calibrated model performance is important, but we have also identified the importance of accurate, temporally resolved LAI. Figure 4 and the sections that follow examine the calibrated results at each site within the 2002 the growing season. Although no species specific LAI observations are available, simulated seasonal LAI values are provided for each species modeled within the stand to confirm that species seasonality and LAI dynamics are physically plausible for that species as well as relative to other modeled stand species.

#### 3.2.1. Appomattox

Appomattox serves as the calibration site for Loblolly Pine, White Oak (*Quercus alba*), Red Maple (*Acer rubrum*) and Sweetgum (*Liquidamber styraciflua*). Normalizing the observed species densities for the four EPIC species subset upward to the observed total stand density approximates within-stand competition for resources at each experimental location. Although the plot description indicates that Loblolly Pine reaches economic maturity i.e., the point when value increase no longer meets or exceeds net return, in 20 years, biological maturity i.e., maximum merchantable volume, occurs later. Literature suggests an average maturity age of about 40 years [38]. Calibration at this site suggests a slightly longer time to maturity (i.e., 55 years) (Table 4).

**Figure 5:**
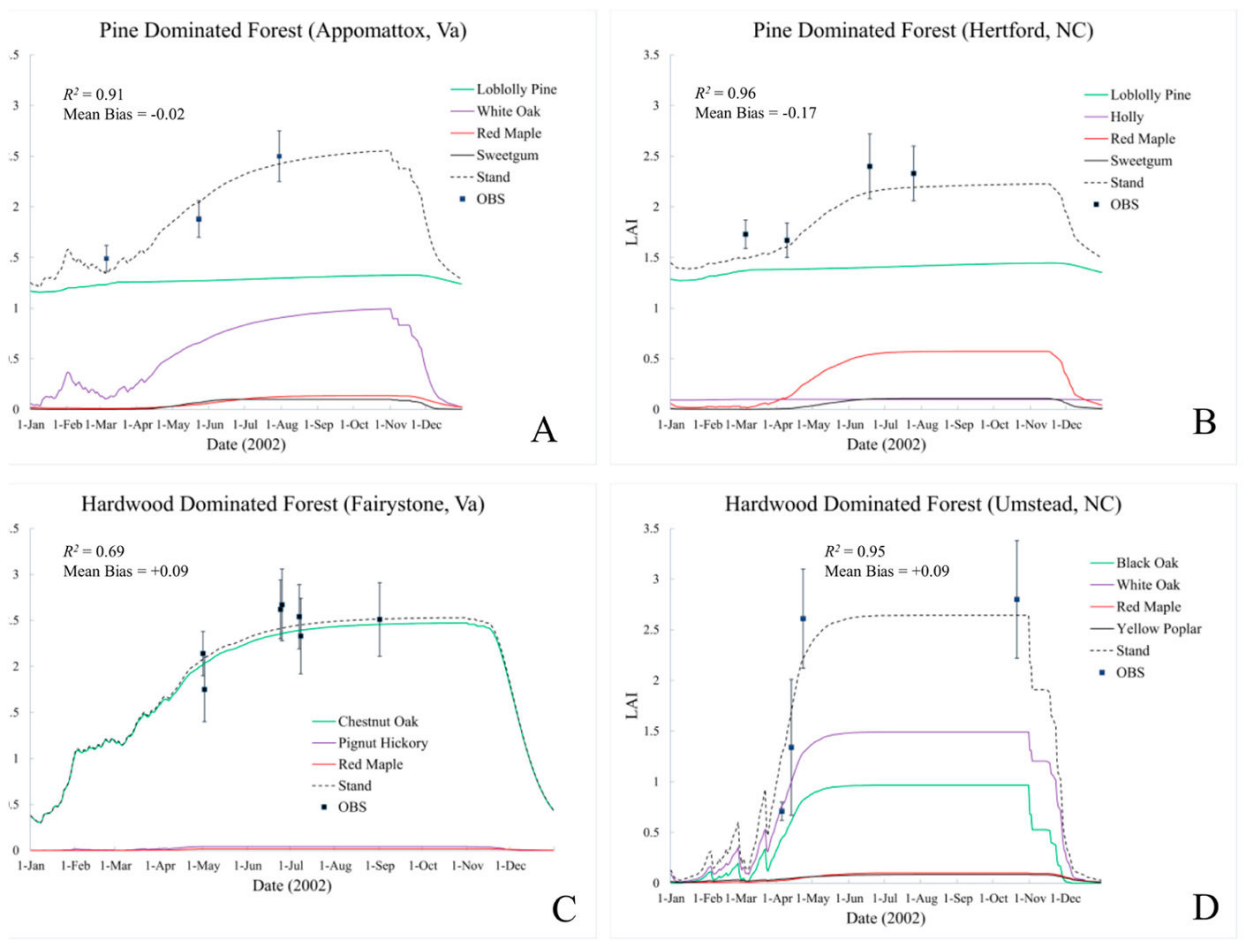
EPIC simulation results at (**A**) Appomattox stand year 19, (**B**) Hertford stand year 20, (**C**) Fairystone stand year 80, and (**D**) Umstead stand year 80. Error bars represent ±1 standard deviations across experimental plot data. *R*^2^ is the simulated and observed data correlation and MB is the mean bias. (Note: ‘OBS’—observed)

Figure 5A summarizes the simulation results for Appomattox. Black squares indicate the mean observed stand-level LAI across samples within the quadrat (Table 3). The whiskers represent one standard deviation (±1 STD) across samples. Loblolly Pine dominates overall LAI. Comparison of observed and simulated LAI indicates a small negative bias (−0.019), and an *R*^2^ value of 0.91. Simulated canopy height is biased 3% high.

A cold temperature damage function reduces EPIC LAI when the minimum daily temperature lies below −1 °C. White Oak early season LAI losses reflect damage from low temperature events following a period of mean temperatures exceeding the White Oak growth threshold (2 °C). EPIC parameters define two points on a frost damage curve. The calibrated white oak damage function predicts that 10% of LAI is lost for each day the minimum temperature is −5 °C, and 50% LAI loss for each day the minimum temperature is −15 °C. Loss estimates change linearly between these two limits, and are more likely to understate frost damage than to overstate it [33]. In contrast, calibrated Loblolly Pine parameters suggest only a 1% per day LAI loss at −5 °C and a 3% per day LAI loss at −15 °C. There are several freeze damage events during the month of February, and an isolated single day damaging freeze event on March 23 (Figure 3).

#### 3.2.2. Hertford

Hertford (Figure 5B) serves as the calibration site for American Holly (*Ilex opaca*), and a verification site for Loblolly Pine, Red Maple and Sweetgum. Although Hertford appears to experience some of the coldest spring temperatures of the four sites (Figure 3), Loblolly Pine is relatively cold tolerant. The stand level EPIC LAI estimates lie within ±1 STD of the field observations, are biased slightly low (−0.17) and have an *R*^2^ value of 0.96. Dominant species (Pine) canopy height is biased 5% high.

#### 3.2.3. Fairystone

Fairystone serves as a verification site for Red Maple and a calibration site for Chestnut Oak (*Quercus prinus*) and Pignut Hickory (*Carya glabra*). Calibrated results are shown in Figure 5C. Chestnut Oak completely dominates the stand LAI. Early LAI variability indicate a series of freeze events during February and March. Chestnut Oak is relatively sensitive to low temperatures and, with its extreme dominance as regards stand LAI, there is significant variability in the early stand-level LAI. Model correlation is the poorest here as compared to the other sites, with simulated LAI *R*^2^ of 0.69, but mean bias is quite good at 0.09 and canopy height of 16.33 m falling well within the reported range for this site (14.6–22.1 m). Examination of plot Figure 5C illustrates one shortcoming of this simulated approach which is an inability to capture unusual events beyond heat, cold and excessive or insufficient moisture. In this case, there is an observed anomalous LAI drop between the 25 June and 8 July observations. Further investigation indicates several 2 to 4 cm precipitation events during this period suggesting the potential for localized leaf loss associated with high wind or hail. Our simulation model is unable to account for these kinds of episodic losses.

#### 3.2.4. Umstead

Umstead serves as a verification site for White Oak and a calibration site for Yellow Poplar (*Liriodendron tulipifera*), Black Oak (*Quercus velutina*) and Northern Red Oak (*Quercus rubra*). Calibrated results are shown in Figure 5D. The oak species are the primary contributors to stand level LAI. Like the Appomattox and Fairystone sites, there are indications of late winter, early spring stress that temporarily reduces leaf area. Stand level EPIC estimates fall within ±1 STD of the observations with the exception of 23 April, produce a mean bias of 0.09 and an *R*^2^ value of 0.95. Canopy height is biased 9% high.

## 4. Discussion

While the results presented in Section 3 suggest EPIC has an ability to simulate mixed forest stand LAI at the experimental plot scale, and previous EPIC applications have been performed for small watersheds, regional air quality models require simulations for much larger areas for which detailed information regarding stand characteristics needed by the EPIC model is often lacking. In addition, both experimental sites and SWAT land units often reflect relatively even-aged stands, i.e., the site is cleared at the start of an experiment. This will not be the case on a larger, regional basis and unmanaged or minimally managed stands. The discussion that follows illustrates one means of addressing these challenges.

The EPIC model requires information regarding species, species density and species age. It then uses this information to model a single, homogeneous mixture of competing forest species. Neither characterization is likely to precisely represent any “real world” natural stand, but can serve to bound the range of possible stand-level LAI estimates. Both areal coverage and species density are rarely available for the same experimental site, but the US Forest Service Forest Inventory and Analysis (FIA) plot calculator (http://apps.fs.fed.us/Evalidator/evalidator.jsp) can provide a consistent, reproducible estimate of species area and density information for a ~50 mi^2^ (~130 km^2^) area (4 mi or 6.4 km radius circle) surrounding the Appomattox field site (Table 5). Approximately 57% of the area within this radius is reported as being forested.

The FIA summary data are aggregated into three species groupings: (1) Loblolly Pine and Virginia Pine (*Pinus virginiana*) represented by Loblolly Pine; (2) mixed oak species comprised of Chestnut, Black and Red Oaks, represented by Black Oak; and (3) mixed upland hardwoods, represented by Red Maple. Stem densities for the groupings and stem fraction in trees per hectare (TPH) are 1349 (76%), 90 (5%) and 339 (19%). This compares to the Appomattox experimental plot represented by Loblolly Pine (26%), White Oak (34%), Red Maple (37%) and Sweetgum (3%). Stand densities are 1778 TPH for the FIA area stand compared to 4856 TPH for the Appomattox experimental plot. The FIA species age distribution suggests a 60-year simulation period with Black Oaks “planted” in year 1 (1942) of the simulation, Red Maples “planted” in year 20 (1961) of the simulation and Loblolly Pine “planted” in year 40 (1981) of the 60-year simulation. The FIA summary suggests relatively even ages within each species. If this were not the case, a range of ages within a species could be simulated by introducing (“planting”) new saplings each year within a desired establishment window. With some simplification, this use of FIA data is similar to that described for boreal forests [29]. For instance, this research introduces a temporally dynamic function to simulate population dynamics that is missing from our example [29] and other research suggests the use of temporally variable growth parameters to represent rapidly developing or “short-rotation” species [39].

**Figure 6:**
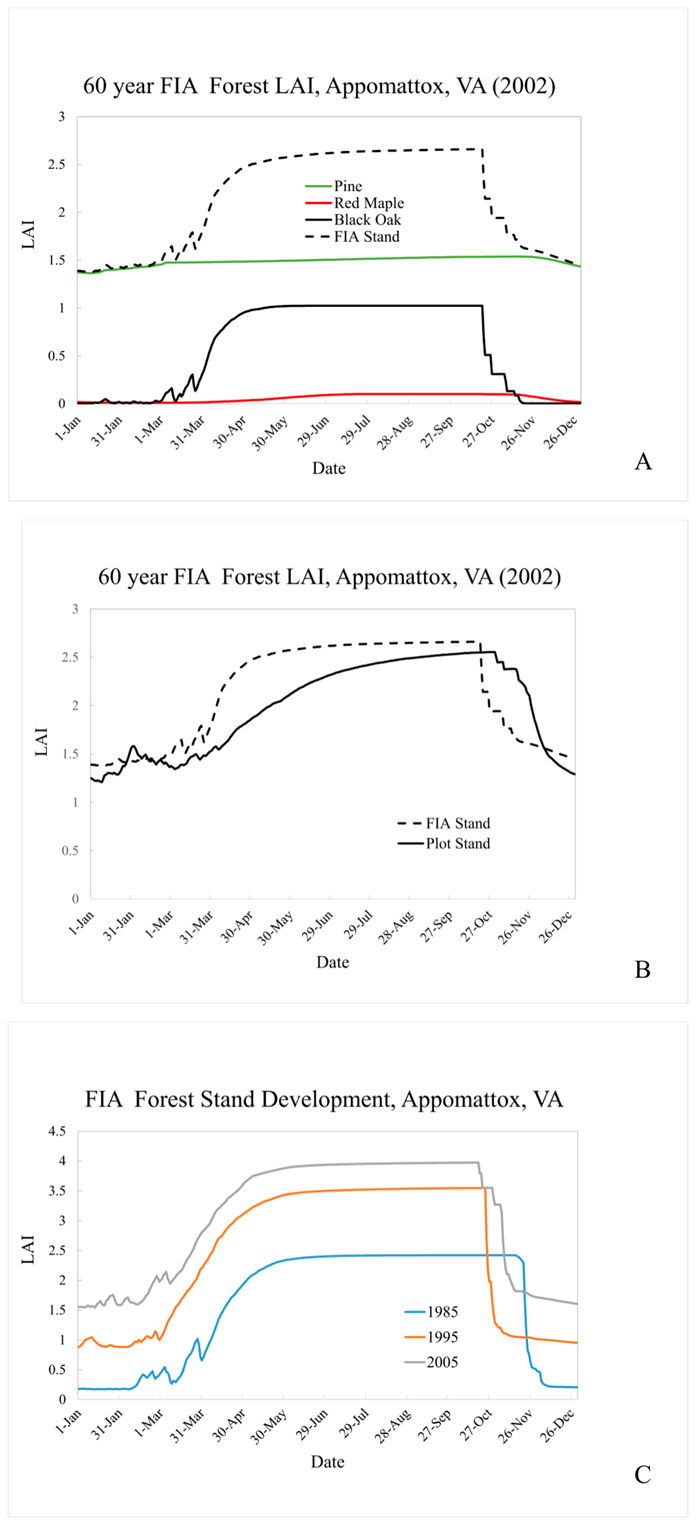
EPIC simulation results for (**A**) FIA stand characterization for the area surrounding the Appomattox, VA experimental plot, (**B**) a comparison of FIA area and plot-level stands and (**C**) FIA stand characterization for the area surrounding Appomattox, VA 1985 through 2006.

Figure 6A shows simulated LAI for the FIA area surrounding the Appomattox field site. For 2002 conditions, the substantial number of Pine provides a relatively constant base LAI of 1.5 throughout the winter months. Black Oak LAI provides the bulk of the stand seasonal signature, with green-up well under-way by April 1 and rapid brown-down (senescence) in late October. Figure 6B compares our plot stand LAI in 2002 to our FIA representation. The seasonal peak LAI value is similar across stands, with the FIA stand’s greater maturity compensating for its lower overall stem density. The simulated Pine LAI is similar across the two simulations (plot value of ~1.2 and FIA value of ~1.4). This is, perhaps a bit surprising since pine are introduced into an existing hardwood stand for the FIA simulation but all species are “planted” simultaneously in the plot simulation. This result, however, appears to be supported by [40] who report that hardwoods may facilitate longleaf pine seedling establishment, as opposed to suppressing it, at hardwood densities as high as 1400 stems·ha^−1^. This simulated species interaction is explored further in Figure 6C, which follows the FIA stand development from 1985 through 2005 (post pine introduction). Changes in stand level LAI magnitude and overall seasonal shape over time reflect simulated stand evolution and weather-driven variability. For instance, mean March minimum temperatures increase slightly between 1985 (~1°C) and 2005 (2.4°C). The simulated LAI seasonal pattern is guided by the accumulation of heat units above a species-specific “base” until a fixed annual total is reached. More rapid heat unit accumulation in the spring means that the annual total may be reached earlier in the year, effectively shifting brown-down initiation earlier as well. Changes such as these could be important for regional simulation of air quality since, as mentioned previously, LAI influences pollutant removal through gas phase deposition, and the magnitude and timing of pollutant precursor emissions can influence subsequent pollutant production and destruction.

**Figure 7:**
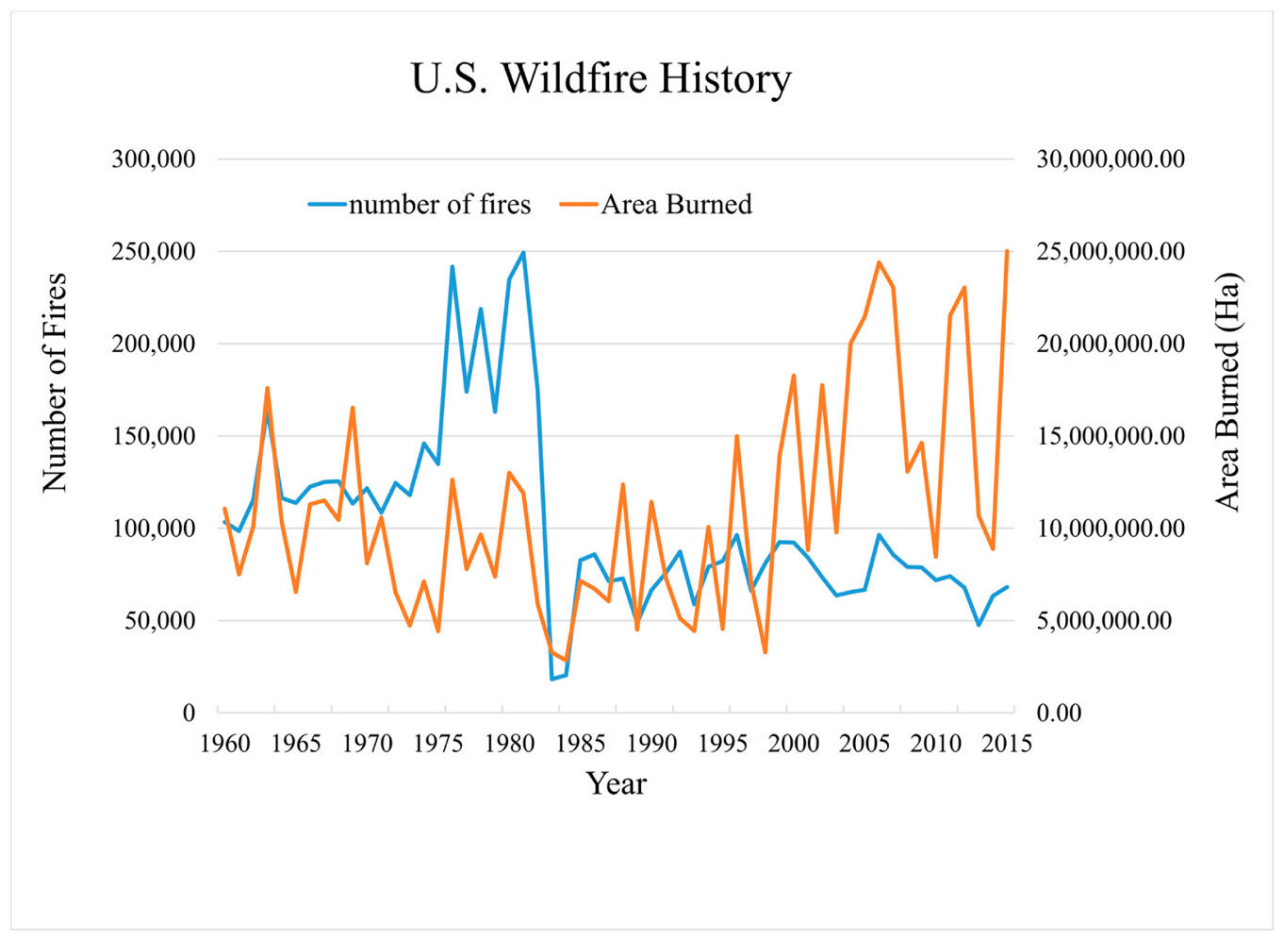
U.S. wildfire history, 1960–2014. Source (https://www.nifc.gov/fireInfo/fireInfo_statistics.html).

An emerging research area to which improved areal LAI estimates could make a significant contribution is episodic land use change associated with wildland fires and prescribed burns used as part of land and resource management (https://www.fs.fed.us/fire/). Figure 7 illustrates the magnitude of the wildfire issue in terms of number of fires and total area burned. Air quality modeling research scientists are responding by explicitly including particulate emissions generated by these fires in their simulations (e.g., [41]). The majority of these fire events are small, but 59 wildfire events, each of which were responsible for burning more than 100,000 acres (247,105 ha) were reported from January 2010 through December 2015. As our ability to simulate these events improves, it becomes important to include associated landscape changes such as surface exposure and forest stand re-growth in our estimates of subsequent pollutant deposition and precursor emission.

EPIC is an attractive option for capturing large-scale interactions between forest land covers and air quality because it is relatively easy to implement and has already been successfully coupled with a regional air quality model [42]. There are, however, other forest ecosystem options that could be considered such as the LANDIS and LANDIS-II models [43,44]. It is always challenging to balance the desire for ecological and biogeochemical realism against the additional resources required for their inclusion. Additional research is needed to determine the level of process detail needed to adequately characterize these complex air and land surface interactions for regional air quality applications.

Finally, modeled LAI has the potential to augment satellite-derived estimates of this same parameter. In a perfect world, satellite estimates would provide reliable LAI in magnitude and without data drops, however this is almost never the case across all forested types and biomes. As an example, the Bigfoot MODIS Validation Project found agreement between validation data and MODIS LAI at low levels of LAI but was problematic at higher biomass levels [45]. Also, they found significant differences dependent on the algorithm pathway chosen, which was a product of atmospheric interference (i.e., cloud contamination) and the number of quality scenes acquired in an 8-day chronosequence. EPIC or any other validated biomass model may work in combination with the satellite feed by constraining or inflating LAI values to reasonable figures based on biases observed in these validation studies [46]. Thus, the modeled LAI could provide bias corrections where the satellite-derived LAI could provide the timing of green-up and senescence and relative seasonal changes in LAI.

## 5. Conclusions

This study has explored the calibration of a semi-empirical, process-based biogeochemical model (EPIC) to estimate stand-level LAI and canopy height in four unfertilized mixed forest sites located in North Carolina and Virginia. Measurements of forest composition (species and number), LAI, diameter (DBH), height and basal area were recorded at each site during the 2002 field season. Calibration and verification results are good at these sites, with modeled to observed LAI correlations ranging from 0.61 to 0.96, correct identification of dominant species evolution, and dominant species (canopy) height estimate within 10% of observation (Table 6). Across all sites and observations, calibration produces an *R*^2^ value of 0.83 and mean bias of +0.008. These results suggest that EPIC offers an LAI estimate that agrees with observations while, at the same time better representing ecosystem processes and dynamic temporal patterns that respond to current and future meteorological conditions. All model calibrations reported here assume a uniform mixture of tree species. This appears to be a reasonable assumption for these stands, but more heavily managed mono-species stands are also easily simulated.

EPIC LAI estimates in regional air quality models such as CMAQ, but additional work is needed to operationalize this approach. For instance, we were able to calibrate and validate only a limited subset of species for a single geographic region. Calibration of additional species and validation across more geographically diverse settings and forest landscapes is needed, e.g., [27]. While application of the EPIC LAI model across the continental U.S., a common CMAQ modeling domain, could require significant resources, this preliminary analysis suggests that it is technically feasible to define general eco-system-level stand composition profiles appropriate for larger geographic or ecological areas. Validation and evaluation of such areal estimates will be needed, but even satellite estimates of areal LAI are prone to error, so that a combination of remotely sensed data and measured atmospheric chemical fluxes may be appropriate. More temporally and spatially detailed characterization of vegetation cover should lead to more realistic atmospheric flux (precursor emission and chemical deposition) and ecosystem exposure estimation. Additional model evaluation is needed in order to better address long-term air quality trends and inter-annual variability that include emerging landscape drivers such as wildland and managed fires.

## Supplementary Material

S1

## Figures and Tables

**Table 1:** Locations of historical weather data sites assigned to each forest site and the distance between the forest and weather data locations.

Stand Site	Lat/Long	Elev. (m)	Coop Site	Lat/Long	Elev. (m)	Dist. (km)
Appomattox	37.22, −78.88	200	Appomattox, VA/51011	37.36, −78.83	277.4	15.8
Hertford	36.38, −77.00	10	Jackson, NC/374456	36.40, −77.42	39.6	37.9
Fairystone	36.77, −80.09	470	Martinsville, VA/515300	36.71, −79.87	231	21
Umstead	35.86, −78.74	94	Raleigh, NC/377079	35.79, −78.70	121.9	8.2

**Table 2:** Annual summary of the number of water, nitrogen and temperature stress days for tree species simulated at the experimental plot during 2002 at the Appomattox field site. Multiple stress conditions may exist for each species on a single day. These counts represent the number of days on which the stress factor was dominate. Temperature stress includes both high and low temperature stresses.

Tree Species	Water (Days)	Nitrogen (Days)	Temperature (Days)
Loblolly pine	39	1	125
Sweetgum	39	1	190
White oak	37	0	208
Red maple	41	0	125

**Table 3:** Observed LAI mean and standard deviation used in EPIC calibration and evaluation. Q indicates the field site quadrat from which the sample is taken (Note: * LAI collection dates after mechanic removal of understory, data not used).

Site	Quad	Date	LAI (Mean)	LAI (Std Dev)
Fairystone	Q1	1-May	2.14	0.24
	Q2	1-May	1.75	0.35
	Q1	25-June	2.62	0.32
	Q2	25-June	2.67	0.38
	Q3	8-July	2.33	0.41
	Q4	8-July	2.54	0.35
	Q4	1-September	2.51	0.4
Umstead	Q1	5-April	0.71	0.09
	Q1	13-April	1.34	0.67
	Q1	23-April	2.61	0.49
	Q1	21-October	2.8	0.58
Appomattox	Q1	6-March	1.49	0.13
	Q1	23-May	1.88	0.18
	Q1	30-July	2.5	0.25
	Q1	6-August *	2.17	0.08
Hertford	Q1	5-March	1.73	0.14
	Q1	9-April	1.67	0.17
	Q1	18-June	2.4	0.32
	Q1	25-July	2.33	0.27
	Q1	5-August *	2.11	0.24

**Table 4:** EPIC species and management information for each simulation location. C = calibration, V = verification, Ch.Oak = Chestnut Oak, Am. Holly = American Holly, Bl Oak = Black Oak, N Red Oak = Northern Red Oak.

	Loblolly Pine	White Oak	Red Maple	Sweetgum	Chestnut Oak	Pignut Hickory	American Holly	Yellow Poplar	Black Oak
**Appomattox**	C	C	C	C					
*Bio Maturity (years)*	55	175	100	60					
Normalized Density (stems)	1246	1655	1805	150					
*Species %*	25.7	34.1	37.2	3					
*Stand Age*	19	19	19	19					
**Hertford**	V		V	V			C		
*Bio Maturity (years)*	55		100	60			100		
Normalized Density (stems)	1482		2862	668			1626		
*Species %*	22.3		43.1	10.1			24.5		
*Stand Age*	20		20	20			20		
**Fairystone**			V		C	C			
*Bio Maturity (years)*			100		150	100			
Normalized Density (stems)			120		307	39			
*Species %*			25.7		65.9	8.4			
*Stand Age*			80		80	80			
**Umstead**		V						C *	C
*Bio Maturity (years)*		175						100	100
Normalized Density (stems)		184						1	86
*Species %*		30.5						0.1	14.3
*Stand Age*		80						80	80

* This species was included to capture available species-specific canopy height observations for this site.

**Table 5:** FIA plot calculator results for the area surrounding the Appomattox plot site, 2008–2013. TPH = Trees per hectare (percent of stand total).

	Timberland (Ha)	TPH	*Age Class*	*Age Class*	*Age Class*

			*1–20 year* %	*21–40 year* %	*41–60 year* %
*Loblolly pine*	14,755	931 (52)	100		
*Virginia Pine*	3726	418 (24)	100		
*Mixed Oak*	12,007	90 (5)			100
*Mixed upland hardwoods*	14,717	339 (19)		100	
***Total Forested***	**18,294**				
***Total Area***	**32,170**				

**Table 6:** Observed and simulated LAI correlation, mean bias and canopy height.

Stand Site	Obs.	LAI *R*^2^	LAI (Mean Bias)	Obs. Dominant Height (m)	EPIC Dominant Species Height (m)
Appomattox	3	0.91	−0.02	15.9	14.9, Loblolly Pine
Hertford	5	0.96	−0.17	14.3	17.1, Loblolly Pine
Fairystone	7	0.69	0.09	14.6–22.1	18.0, Chestnut Oak
Umstead	4	0.95	0.09	12.8	12.3, White Oak
