# Supplementary material for: A Comparison of Simulated and Field-Derived Leaf Area Index (LAI) and Canopy Height Values from Four Forest Complexes in the Southeastern USA
Source: Forests. Author manuscript; Available in PMC 2018 May 16. (PMC5954438; doi:10.3390/f9010026)
Supplement: S1 [file NIHMS960044-supplement-S1.pdf]

forests

MDPI

Supplemental Information

A comparison of simulated and field-derived leaf area index (LAI) and canopy height values from four forest complexes in the southeastern USA

John S. Iiames [1,*], Ellen Cooter [2], Donna Schwede [2] and Jimmy Williams [3]

1 U.S. Environmental Protection Agency, National Exposure Research Laboratory, Exposure Methods and Measurements Division, 109 T.W. Alexander Drive, Research Triangle Park, N.C. 27711; Iiames.john@epa.gov
2 U.S. Environmental Protection Agency, National Exposure Research Laboratory, Atmospheric Modeling and Analysis Division, 109 T.W. Alexander Drive, Research Triangle Park, N.C. 27711; Cooter.ellen@epa.gov; Schwede.donna@epa.gov
3 Texas A & M University, Agri-Life Research, Temple, Texas; jwilliams@brc.tamus.edu
* Correspondence: Iiames.john@epa.gov; Tel.: +1-919-541-3039

1. Field site forest visualization and composition and EPIC model input

1.1 Appomattox

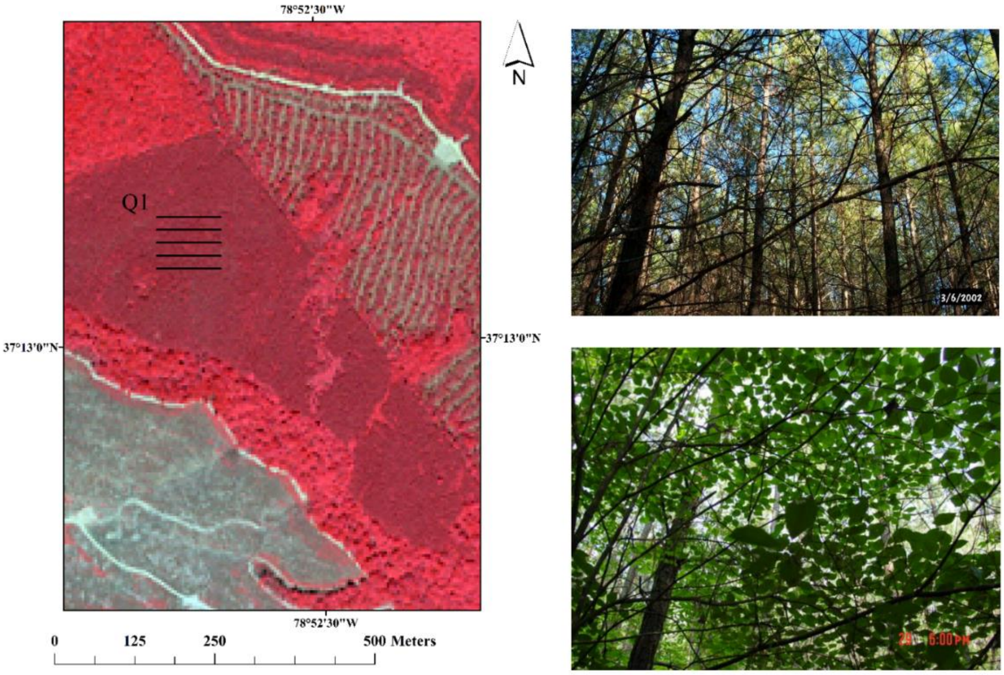

Figure S1. Appomattox, VA site location. Image on left is a color infrared Ikonos image with plot location (Q1) depicted within the loblolly pine stand (dark red tone). Leaf-off and leaf-on images are shown on the right.

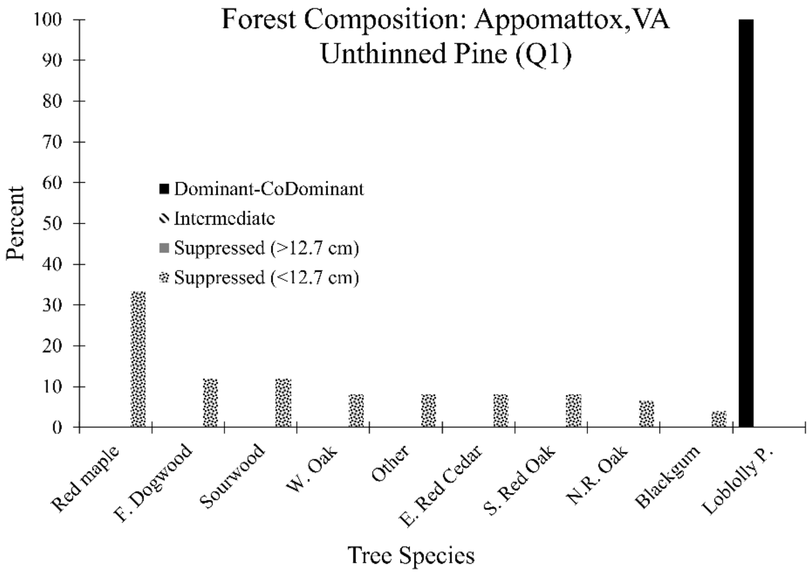

Figure S2. Deciduous forest composition for the Appomattox LAI validation site, dominant-codominant, intermediate, and suppressed canopy.

1.2 Hertford

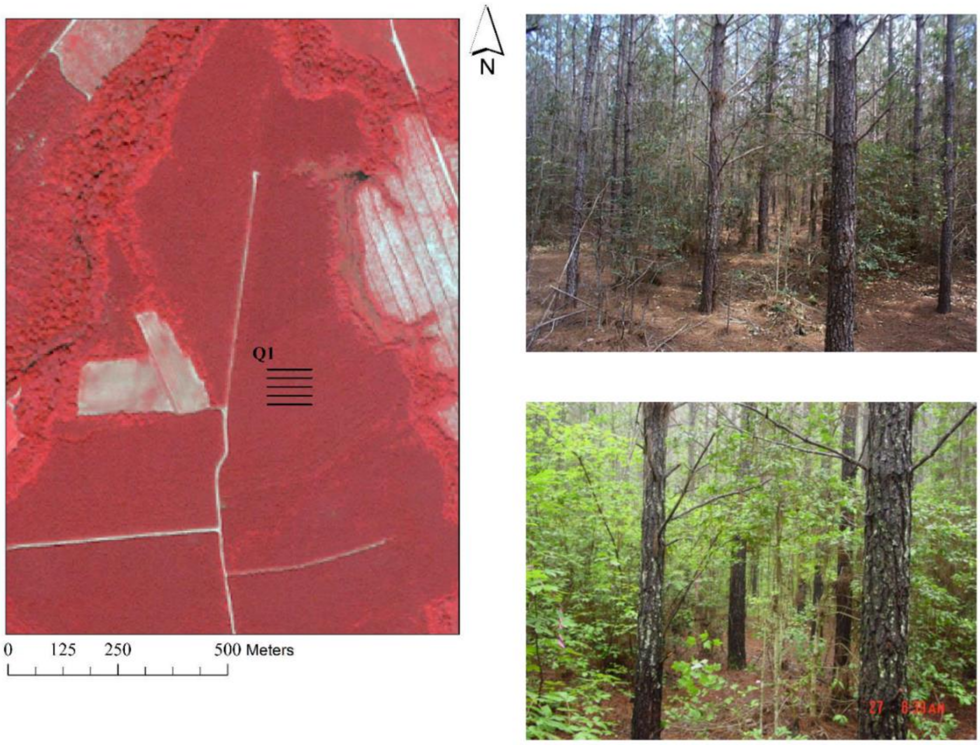

Figure S3 Hertford, NC site location. Image on left is a color infrared Ikonos image with plot location (Q1) depicted within the loblolly pine stand (dark red tone). Leaf-off and leaf-on images are shown on the right.

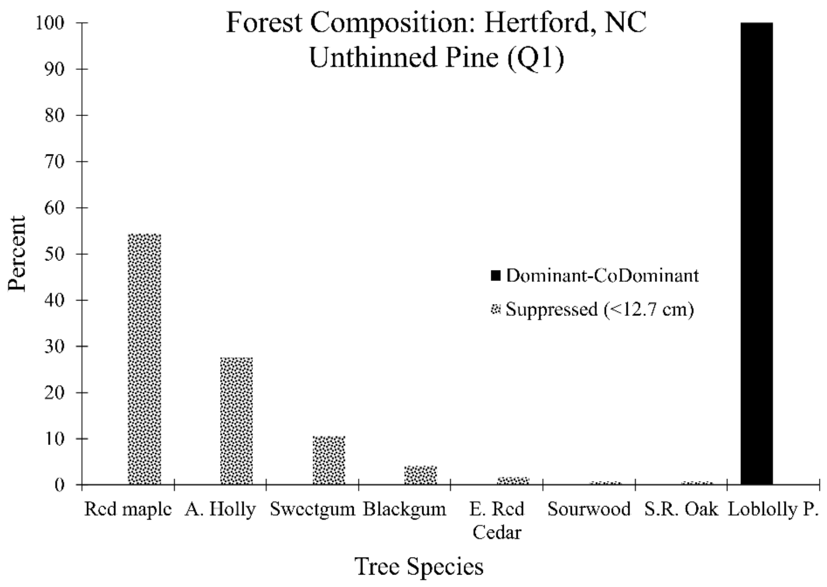

Figure S4. Deciduous forest composition for the Hertford LAI validation site, dominant-codominant, intermediate, and suppressed canopy.

1.3 *Fairystone*

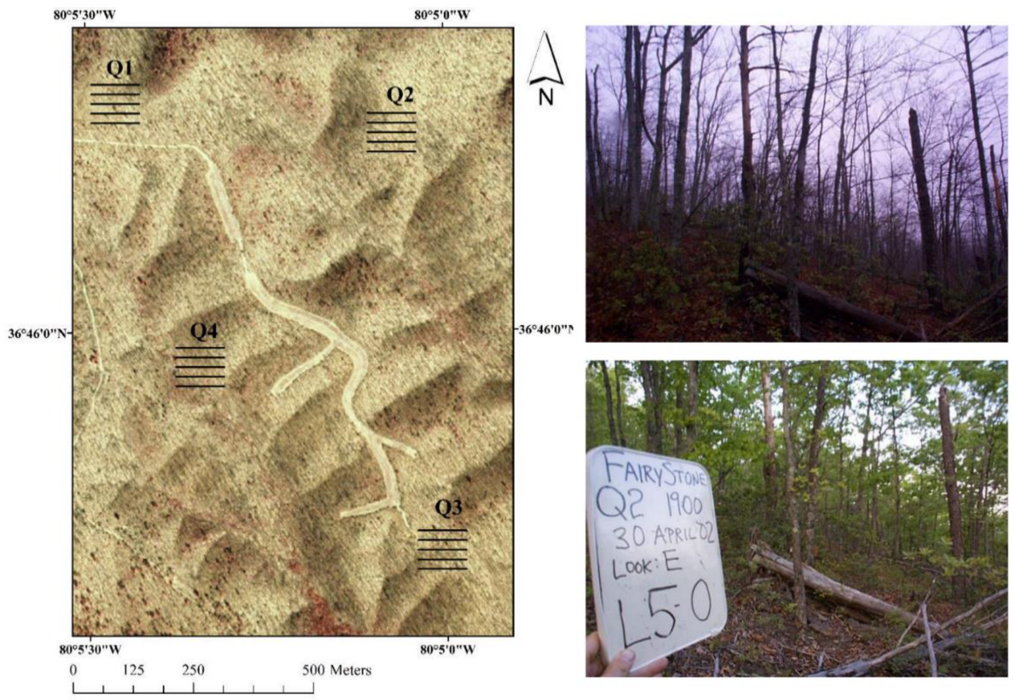

Figure S5. Fairystone, VA site location. Image on left is a natural color digital ortho-quarter quadrangle image with plot locations (Q1-Q4) depicted within the oak-hickory hardwood stand. Leaf-off and leaf-on images are shown on the right.

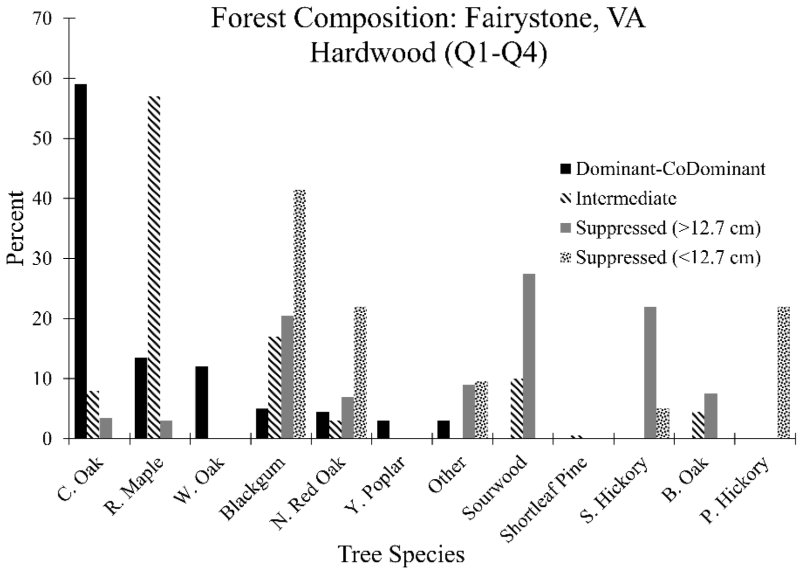

Figure S6. Deciduous forest composition for the Fairystone LAI validation site, dominant-codominant, intermediate, and suppressed canopy.

1.4 Umstead

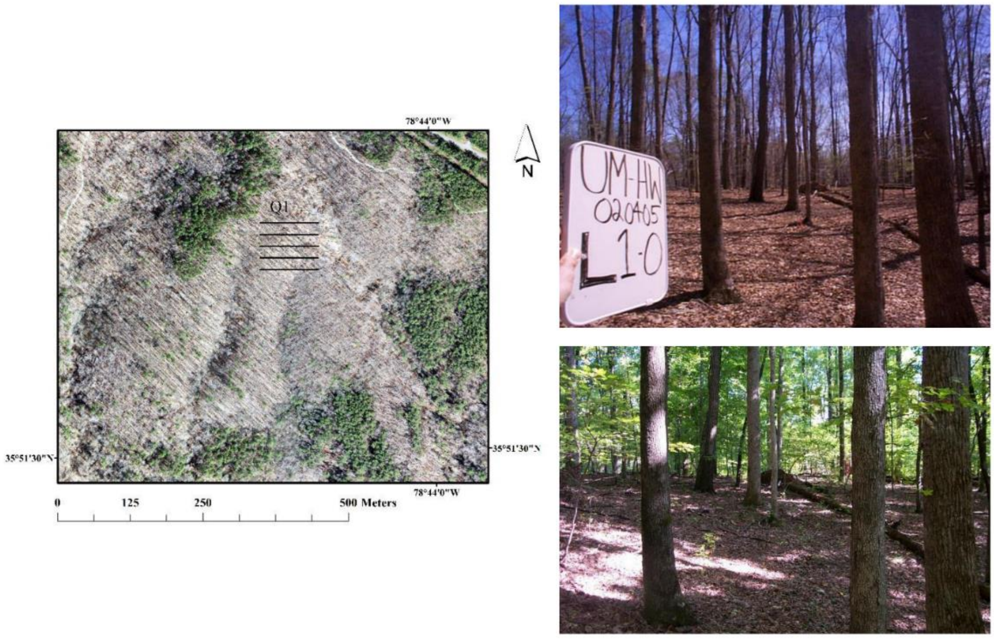

Figure S7. Umstead, NC site location. Image on left is a natural color digital ortho-quarter quadrangle image with plot location Q1 depicted within the oak-hickory hardwood stand. Leaf-off and leaf-on images are shown on the right.

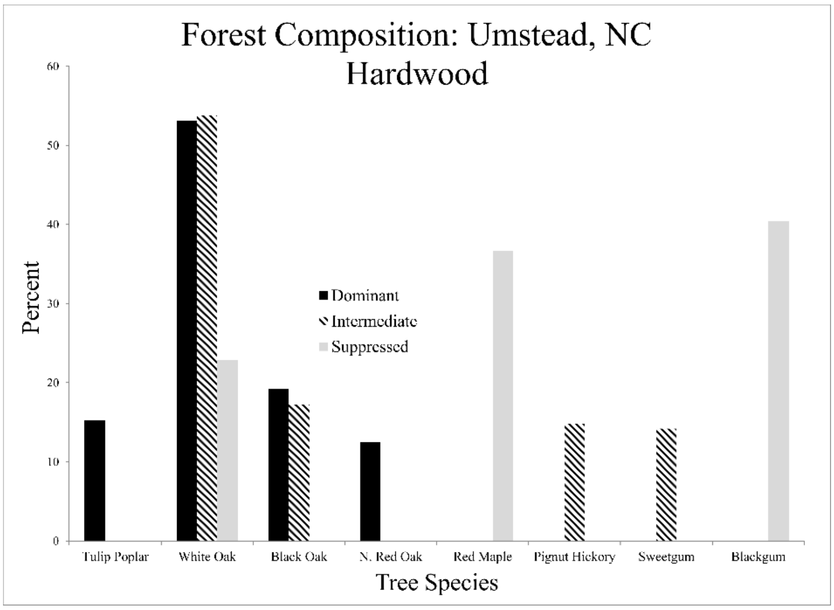

Figure S8 Deciduous forest composition for the Fairystone LAI validation site, dominant-codominant, intermediate, and suppressed canopy.

1.5 All sites composition summary

Table S1. Forest stand structural attributes.

	Appomattox	*Hertford*	*Fairystone*	*Umstead*
TPH (Dom/CoDom)	1250	1740	288.8	101.5
TPH (Intermediate)			459	169.6
TPH (Suppressed)			277.9	333.6
TPH (Understory)	3790	2830		
Stand Age (years)	23	19	80	80
DBH (cm) (Dom/CoDom)			24.8	43.5
DBH (cm) (Intermediate)			11.4	22.9
DBH (cm) (Suppressed)			11.1	11.7
DBH (cm) overall	21.6	18.5		
Height (m) (Dom only)	15.9	14.3	18.9	24.4
BA/H (Dom/CoDom)			12.3	14.8
BA/H (Intermediate)			4.7	7.5
BA/H (Suppressed)			3.7	4.3
BA/H overall	36.7	37.3		
CC%	71	71		

2. EPIC model inputs and model runs

Table S2. Initial Cecil (1292NC0018VAC) soil profile used as input to the EPIC model at all forest calibration and verification sites.

Variable Name	*layer 1*	*layer 2*	*layer 3*	*layer 4*	*layer 5*
depth to bottom of layer (m)	0.1	0.18	0.28	1.26	1.9
bulk density (t m^{-3})	1.54	1.54	1.48	1.48	1.67
wilting point (m m^{-1})	0.06	0.06	0.15	0.27	0.01
field capacity (m m^{-1})	0.13	0.13	0.28	0.42	0.04
% sand	67.85	67.85	55.08	18.14	67.85
% silt	19.65	19.65	17.42	29.36	19.65
soil pH	6.6	6.6	5	5	0
organic carbon conc. (%)	1.56	1.39	1.16	0.12	0.02
cation exch. cpcty (cmol kg^{-1})	3.82	3.82	5.94	5.43	0
coarse fragment content (% vol.)	25	25	0	0	0
initial NO3 concentration (g t^{-1})	5	5	5	2	2
initial labile P concentration (g t^{-1})	8	8	8	4	4
bulk density oven dry (t m^{-3})	1.54	1.54	1.48	1.48	1.67

The single, "representative" soil that was used at all of the forest sites (Table S1.3) derives from soil parameters contained within the Baumer database built by Dr. Otto Baumer shortly after he retired from the USDA, National Resources Conservations Service (NRCS) Soils Laboratory in Lincoln, Nebraska. Dr. Baumer created the database under contract with the Texas A&M Blackland Research Station. The EPIC soil datasets were built to represent the sample point soils selected for the 1997 USDA Natural Resources Inventory (NRI) data points. However, the Baumer database does not include complete datasets for all soils sampled by the NRI because some soils lacked key information to build the EPIC soil file. This analysis used soils identified in the Baumer database as complete. Dr. Baumer used the SOILS-5 database (Soils-5 is the name of the input form used to enter data into the Official Series Descriptions for SCS soil surveys) and soil pedon data to develop the representative EPIC data sets.

The Baumer data base includes soil information by state compiled from various sources he acquired. The files contained some information on over 200,000 soils at NRI points. A subset of nearly 45,000 soils contained potentially usable data. This information was used to create a subset of soil parameters to be used with EPIC for almost 23,000 soils. The Baumer soils data base may be downloaded as part of the Fertilizer Emission Scenario Tool for CMAQ (FEST-C) package available at no charge at http://www.cmascenter.org/.

Table S3a. Selected initial EPIC crop parameter values for tree species simulated at the four forest field sample sites. An entry of N/A indicates no initial parameter values were available

	Pine	***Oak***	***R. Maple***	***Swt. Gum***	***Ch. Oak***	***P. Hickory***	***A. Holly***	***Y. Poplar***	***B. Oak***	***NR Oak***
WA	16	15	N/A	16	N/A	N/A	N/A	30	N/A	N/A
TOP	20	30	N/A	25	N/A	N/A	N/A	30	N/A	N/A
TBS	2	10	N/A	5	N/A	N/A	N/A	10	N/A	N/A
DMLA	5	5	N/A	5	N/A	N/A	N/A	5	N/A	N/A
DLAI	0.15	0.99	N/A	0.75	N/A	N/A	N/A	0.99	N/A	N/A
DLAP1	10.5	5.05	N/A	15.4	N/A	N/A	N/A	5.05	N/A	N/A
DLAP2	25.99	40.95	N/A	30.8	N/A	N/A	N/A	40.95	N/A	N/A
RBMD	1	1	N/A	1	N/A	N/A	N/A	1	N/A	N/A
HMX	20	6	N/A	80	N/A	N/A	N/A	7.5	N/A	N/A
FRST1	5.01	5.1	N/A	5.1	N/A	N/A	N/A	5.1	N/A	N/A
FRST2	15.03	15.5	N/A	15.5	N/A	N/A	N/A	15.5	N/A	N/A
PPLP1	1000.95	1000.95	N/A	1000.95	N/A	N/A	N/A	500.95	N/A	N/A
PPLP2	100.1	100.05	N/A	100.05	N/A	N/A	N/A	20.15	N/A	N/A

Table S3b. Selected calibrated EPIC crop parameter values for tree species simulated at the four forest field sample sites.

	Pine	***Oak***	***R. Maple***	***Swt. Gum***	***Ch. Oak***	***P. Hickory***	***A. Holly***	***Y. Poplar***	***B. Oak***	***NR Oak***
WA	16	15	15	16	15	15	16	30	15	15
TOP	20	30	20	25	30	20	20	30	30	30
TBS	2	10	2	10	10	2	5	10	10	10
DMLA	0.5	3	3.75	3	5	4	2	6	3	5.5
DLAI	0.75	0.9	0.5	0.03	0.99	0.05	0.75	0.9	0.9	0.9
DLAP1	5.05	15.7	5.05	10.01	15.7	5.3	5.05	5.2	15.7	15.7
DLAP2	85.95	30.99	40.95	40.95	30.99	30.95	85.95	40.95	30.99	30.99
RBMD	1	1	1	1	0.2	1	1	1	1	1
HMX	23	20	20	20	18	18	7	10	20	20
FRST1	5.01	5.3	5.01	5.01	5.1	5.1	5.01	5.1	5.3	5.3
FRST2	15.03	15.6	15.95	15.95	15.5	15.5	15.03	15.5	15.6	15.6
PPLP1	1500.95	1500.96	1000.95	1000.95	9000.95	9000.95	6000.95	5000.95	1500.95	1500.95
PPLP2	5.05	5.05	300.05	300.05	1500.05	1500.05	2000.05	300.05	5.05	5.05

Table S3c EPIC variable Key (https://www.nrcs.usda.gov/Internet/FSE_DOCUMENTS/nrcs143_012924.pdf)

Variable	Description
WA	Biomass-Energy Ratio, potential unstressed growth rate (kg/MJ)
TOP	Optimal temperature for plant growth (C^o)
TBS	Minimum temperature for plant growth (C^o)
DMLA	Maximum potential leaf area index
DLAI	Fraction of growing season when leaf area declines
DLAP1	First point on optimal leaf area development curve
DLAP2	Second point on optimal leaf area development curve
RBMD	Biomass-energy ratio decline rate parameter
HMX	Maximum crop height
FRST1	First point on frost damage curve
FRST2	First point on frost damage curve
PPLP1	Plant Population for Crops and Grass - 1st Point on curve
PPLP2	Plant Population for Crops and Grass - 2nd Point on curve

References for supplemental data

Bash, J.O.; Cooter, E.J.; Dennis, R.L.; Walker, J.T.; Pleim, J.E. Evaluation of a regional air quality model with bidirectional NH3 exchanged coupled to an agro-ecosystem model. Biogeosciences, 2013, 10, 1635-1645, Available online: https://www.biogeosciences.net/10/1635/2013/ (accessed on 31 October 2017).
